# As soon as there was life, there was danger: the deep history of survival behaviours and the shallower history of consciousness

**DOI:** 10.1098/rstb.2021.0292

**Published:** 2022-02-14

**Authors:** Joseph E. LeDoux

**Affiliations:** Center for Neural Science, New York University, New York, NY 10003, USA

**Keywords:** fear, consciousness, evolution, survival circuits

## Abstract

It is often said that fear is a universal innate emotion that we humans have inherited from our mammalian ancestors by virtue of having inherited conserved features of their nervous systems. Contrary to this common sense-based scientific point of view, I have argued that what we have inherited from our mammalian ancestors, and they from their distal vertebrate ancestors, and they from their chordate ancestors, and so forth, is not a fear circuit. It is, instead, a defensive survival circuit that detects threats, and in response, initiates defensive survival behaviours and supporting physiological adjustments. Seen in this light, the defensive survival circuits of humans and other mammals can be conceptualized as manifestations of an ancient survival function—the ability to detect danger and respond to it—that may in fact predate animals and their nervous systems, and perhaps may go back to the beginning of life. Fear, on the other hand, from my perspective, is a product of cortical cognitive circuits. This conception is not just of academic interest. It also has practical implications, offering clues as to why efforts to treat problems related to fear and anxiety are not more effective, and what might make them better.

This article is part of the theme issue ‘Systems neuroscience through the lens of evolutionary theory’.

## 1. Introduction

That behaviour and evolution are interrelated is hardly a novel idea. Darwin emphasized it, as did pioneering ethologists such as Niko Tinbergen and Konrad Lorenz. The behaviourists who dominated psychology in the first half of the twentieth century paid little attention to evolution, but most contemporary psychologists and neuroscientists accept it as a key factor that must be accounted for to explain behaviour.

Efforts to understand the evolution of behaviour in neuroscience often compare closely related groups, such as humans and other mammals, or even other vertebrates. There are obvious reasons for doing so. For example, since the brain controls behaviour, studies of how brains evolved can help shed light on how behavioural repertoires evolved. But there are also reasons to look deeper [1,2].

I have spent much of my career working on the brain mechanisms of emotion, and especially how the brain detects and responds to danger. Roughly a decade ago, I began to see my work in a new light. This transformation is summed up by the title of the present article, which is a paraphrase of a quote from the American essayist and poet, Ralph Waldo Emerson [3]. In my version of Emerson's comment, I changed both instances of ‘is' to ‘was’. This stems from the realization that I had come to a few years ago—that danger must be as old as life itself. This epiphany may seem obvious to those who have thought long and hard about evolution. But it was an eye-opener to the mammalian neuroscientist in me, and prompted me to explore the natural history of danger in more detail, which I did in my 2019 book, *The deep history of ourselves: the four-billion-year story of how we got conscious brains* [2]. Here, I summarize the path that took me to the science of danger in the first place, and why the latter led me to write *The deep history of ourselves*. I also consider the implications for our understanding of our emotions and their maladies.

## 2. Finding my way to danger

In the late 1970s, I completed my PhD studying consciousness in split-brain patients. From this research, my mentor, Michael Gazzaniga, and I concluded that an important feature of human consciousness is the maintenance of a sense of mental unity via cognitive interpretations that attribute meaning and cause to behaviours that are controlled non-consciously by the brain [4,5]. We speculated that emotional systems might be examples of systems that non-consciously generate behavioural responses, compelling some sort of interpretive narration to sustain a sense of mental unity. The fact that not much was known about emotions at the time made it an attractive option for me, given a comment made by another professor, the comparative anatomist, Harvey Karten, in a graduate seminar on the vertebrate brain. Karten suggested that students should avoid popular research topics, such as the role of the visual cortex in perception or the hippocampus in memory. We should, instead find an interesting but less well-travelled scientific road.

After graduate school, I therefore turned to the brain mechanisms of emotion. Because the tools available for studying the human brain were quite limited at the time, I chose to study how the brain controls emotional behaviours in rodents.

It's not as if the brain mechanisms of emotional behaviour had never been researched. Emotion was actually a popular scientific topic among physiologists in the first half of the twentieth century [6–11]. But when the field of neuroscience emerged as an independent discipline in the 1970s, cognition was the rage, and brain researchers were more interested in the mechanisms underlying processes such perception, attention and memory, than in emotion.

Although the earlier research had laid an important foundation for understanding the emotional brain, the knowledge was piecemeal, and mostly focused on what brain areas do, as if functions were literally localized in areas. The split-brain studies had taught me to think of information flow through the brain, and my ambition was to map the information flow underlying emotional behaviour from sensory receptors to motor outputs. Invertebrate researchers, such as Eric Kandel, had achieved this kind of sensory to motor mapping of behaviour in the invertebrate nervous system [12], and I thought that new research tools becoming available might make this doable in the brains of mammals as well.

## 3. Circuit busting fear conditioning

I adopted the behavioural procedure called Pavlovian fear conditioning [13] (this was also one of the tools Kandel had used). I was not particularly interested in fear, but the fear conditioning paradigm seemed to have some useful properties for studying emotional behaviour. For one thing, its associative underpinnings are the bread-and-butter mechanism by which organisms learn about stimuli that co-occur with biologically significant events. The result is that the previously meaningless stimuli come to trigger species-typical behavioural and physiological responses, allowing the organism to respond to dangerous stimuli in advance of their harmful consequences. Also, the responses elicited in lower mammals are similar to those expressed in humans when in harm's way. Further, much was known about the behavioural principles underlying Pavlovian learning. And finally, consistent with Karten's advice, next to nothing was known about the neural mechanisms in mammals.

I submitted a grant proposal to map emotional behaviour pathways in the rat brain using the Pavlovian paradigm, but it was not funded because ‘emotion is not a neuroscientific topic’. The way I ended up getting funding was by re-framing my work as being about the neural basis of learning.

To map the connections of behavioural and physiological responses that were elicited by Pavlovian conditioned stimuli, I used axonal transport pathway tracing (which I had picked up while doing a rotation in Karten's lab), as well as some other methods that I learned ‘on the job’, such as chemical lesion approaches and electrophysiological recording techniques, especially in freely behaving animals.

I recall a Society for Neuroscience poster session in the mid-1980s where there were hundreds of presentations on the hippocampus and memory, and very few (maybe three or four) on the brain mechanisms of fear conditioning. Although our ranks were small [14–16], within a few years, we fear conditioning researchers had shown that sensory inputs to, and motor outputs from the amygdala are responsible for the behavioural and physiological expression of conditioned fear responses. And importantly, within the amygdala, a systematic pattern of synaptic connectivity from the input region (i.e. lateral nucleus) to the output region (i.e. central nucleus) was revealed. Further, while synaptic plasticity was found to occur throughout the amygdala circuitry, plasticity in the lateral amygdala seemed particularly important, based on the short latency of the neural changes and their necessity in supporting plasticity in other areas. Summaries of this body of work appeared in a 1994 article I wrote for *Scientific American* [17], in my 1996 book, *The emotional brain* [18] and in an *Annual Review of Neuroscience* paper in 2000 [19].

By the late 1990s, many researchers were becoming interested in the neural mechanisms of Pavlovian fear conditioning. With the basic circuits outlined, the effort was turning to the molecular mechanisms that might underlie learning. Some of the new researchers were from the usual areas of psychology and brain research, but others were from genetics, where the ability to alter genes was emerging. Over the next decade or so, accumulated findings implicated specific neurotransmitter receptors, ion channels, protein kinases and macromolecules in learning and plasticity in the amygdala of rodents [20–23]. Many of these findings were guided by earlier results about learning and memory storage in invertebrates [24,25], a point that will become relevant below.

## 4. Three views of fear

As I had hoped when I started this work, the fear conditioning procedure turned out to be an excellent way to elucidate how behavioural and physiological responses elicited by emotional stimuli are learned and controlled non-consciously by the brain. To emphasize my idea that amygdala circuits processed information non-consciously, I adopted the implicit/explicit distinction popular in learning and memory research [26]. Specifically, I referred to the amygdala as an implicit processor that controls fear behaviours non-consciously, with the explicit conscious experience of fear being a product of cortical cognitive circuits [18].

Findings obtained in studies of humans provided support for the idea that the amygdala non-consciously controls behavioural and physiological responses elicited by threats, turning one of my split-brain ideas into a reality. For example, subliminal presentation of threats resulted in amygdala activation and elicited physiological responses without the person having any awareness of the stimulus or reporting any feeling of fear [27,28]. Later studies showed that even when pressed, healthy participants did not report emotional feeling states in response to subliminal emotional stimuli [29]. A related line of research showed that in the so-called blindsight patients threats also elicited amygdala activity and body responses without conscious awareness of the stimulus [30]. And studies of patients with amygdala damage showed that they could still, under some conditions, have emotional experiences [31,32]. Fear, it seems, is not inextricably tied to the amygdala in humans. The amygdala, in other words might contribute to, but does not determine, the mental state of fear.

Most researchers working on fear conditioning came to this topic from a behaviourist intellectual background. I was once asked by one such colleague, ‘Why do you talk about emotion?’ He said ‘we just study behavioural learning’. Given this, why did behaviourists ever call the procedure ‘fear’ conditioning?

Although the behaviourists had banned subjective states as explanations of behaviour, they did not eliminate the use of subjective state words in explaining behaviour. They referred to the task as ‘fear’ conditioning because fear for them was a description of the functional relationship between a dangerous stimulus and defensive behaviour. And when some behaviourists became physiological psychologists in the 1950s, the functional relation came to be thought of as a physiological state, which they called ‘fear’, that connected the threatening conditioned stimulus to the defensive conditioned response. The amygdala, of course, became the home of that that mindless fear state [14–16,33,34].

But some researchers rebelled against the sterile conception of behaviour offered by behaviourist approaches [35–40]. The emotion researchers among them promoted the idea that rodent fear was more or less like human fear, and therefore that animal studies could be used to understand human emotions. These anti-behaviourist brain researchers treated subcortical brain areas, like the amygdala and periaqueductal gray, as being responsible not just for defensive behaviours but also for the subjective feeling of fear in animals and humans alike [35].

Both groups of researchers, in other words, used the term ‘fear’ to describe functions subserved by subcortical circuits, but they used the term in different ways. The behaviourists–learning crowd did not bother to explain what they meant. For them, conscious fear was a useless construct that had been successfully purged from scientific discourse, and any serious scientist knows that ‘fear’ is simply a physiological state that controls behaviour. The anti-behaviourists, on the other hand, made it very clear that fear was conscious fear. The general public, and even other scientists who were not ‘in the know’, did not realize the difference, and simply assumed that when a scientist wrote or said ‘fear’ they meant conscious fear. By failing to explicitly define their terms, the behaviourist crew's message was lost.

**Figure 1.**
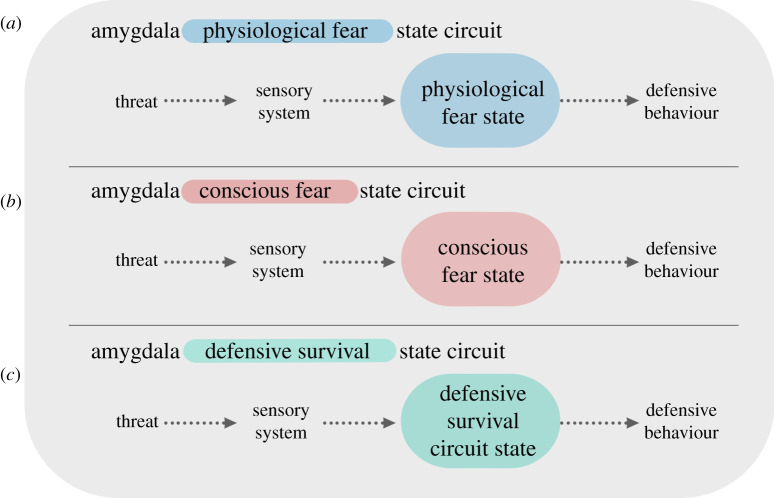
Amygdala fear versus defensive survival circuit views. (Online version in colour.)

I was caught in the middle [18,19,41–43]. I was aligned with my behaviourist-oriented colleagues in emphasizing that physiological states within amygdala circuits control defensive behaviour rather than make fearful feelings. But like the anti-behaviourists, I treated conscious fear as real and important. Yet, I viewed conscious fear as a product of cognitive processes instantiated in cortical circuits, rather than as a hard-wired function of subcortical areas [18,41,42] (figure 1).

## 5. Rethinking the emotional brain

Over the years, the amygdala went from being an obscure brain area of interest to only a handful of neuroscientists to cultural meme—the ‘amygdala fear centre’. This meme appeared in novels, movies, songs, self-help books, cartoons and online merchandise from tee-shirts to amulets to essential oils. Despite my efforts to draw a distinction between conscious fear and its behavioural and physiological correlates, my work was often used as scientific support for the meme, and I was often introduced at scientific and lay lectures as having discovered how conscious feelings of fear arise from the amygdala.

By 2012, it was clear to me that the loose, casual use of common language terms, especially psychological terms, as names for scientific constructs, can have a negative impact on our understanding of scientific findings and their implications. To be perfectly honest, though, I was not always as clear as I now realize I should have been. I resolved to find a better way to write and talk about fear and the brain, both to other scientists and to lay audiences.

I published scientific articles with titles like, ‘Rethinking the emotional brain’, ‘Coming to terms with fear’ and ‘Semantics, surplus meaning and the science of fear’ [44–46], and books called *Anxious* [47] and *The deep history of ourselves* [2]. In these publications, I argued that the use of mental state terms, like ‘fear’, to describe circuits that control behavioural and physiological responses conflates correlation with causation. For example, fear often occurs when we freeze or flee in a dangerous situation, but not because a feeling of fear that bubbles up out of the amygdala is the underlying cause of the responses. Although the subjective experience and the objective responses are both triggered by the same threat stimulus, I propose that they are separate consequences mediated by different outputs of the sensory system. One output takes the threat stimulus information to the amygdala to control behaviour and physiology, and the other takes it to cortical cognitive circuits as part of the process of assembling the conscious state we know of as fear.

In these publications, I emphasized that scientists should strive for conceptual clarity, and I offered a simple solution in the case of emotions. I suggested that we reserve the use of mental state terms, like ‘fear’, for the mental state that the word names, as opposed to using such terms to describe behavioural and physiological correlates of the conscious experience of fear. For example, rather than treating amygdala circuits activated by threats as ‘fear’ circuits of either kind (physiological or subjective), I proposed that they be called ‘defensive survival circuits' (figure 1). After all, they control both defensive behaviours and the physiological responses that provide metabolic support for the behaviours in dangerous situations. I also suggested that the procedure called ‘fear conditioning’ be referred to as ‘threat conditioning’.

The fact is that ‘threat’ and ‘defense’ were already commonly, though inconsistently, used in the field. All I did was suggest that we use the psychologically more neutral terms, instead of the one's with subjective implications, when referring to behaviour and body physiology, saving ‘fear’ for the mental state. There has been some progress, but also resistance. A colleague wrote a commentary about one of my papers [48] saying that following the ideas I have been promoting would take psychiatry back to the dark time when subjectivity ruled the field [49]. As I will argue below, though, the marginalization of subjective experience is why the treatment of mental disorders is not more effective.

## 6. The language of psychology

I am hardly the first to raise questions about scientific terminology of psychology [1,50–55]. Critics have noted that psychologists tend to be sloppy with words; that category names can create illusions of understanding; and that the use of common language terms as names for scientific constructs can skew our understanding of the underlying processes. For example, calling the amygdala a fear generator [49], regardless of whether a physiological or subjective meaning of fear is intended, infects this brain area with subjective properties that it may well not deserve.

It is especially important to be careful when we extrapolate across species based on similarity in behaviours in humans and other animals. When we do this, we are using our introspections about the relation of our mental states to our behaviours to explain animal behaviour. The quote below from the pioneering ethologist, Nico Tinbergen, succinctly summarized the problem some 70 years ago:

‘Hunger, like, anger, fear, and so forth, is a phenomenon that can be known only by introspection. When applied to another … species, it is merely a guess about the possible nature of the animal's subjective state’. [56, p. 5]

Being careful about the use of common language terms as names for scientific constructs is more important in the psychological sciences, including psychological aspects of neuroscience, than in other areas. For example, no one actually thinks that there is a hedgehog living in the gene of that name. But people do believe that fear lives in the amygdala ‘fear centre’.

I agree with those who propose that the traditional psychological categories used to conceptualize behaviour can be problematic [1,57,58]. But I do not think this applies across the board. Problems especially arise when mental state words derived from human introspection [53] are used to talk about behaviours that do not depend on mental states [2,45–47], both in humans and other animals. In such cases, brain circuits that control such behaviours inherit the mental implications of the common language terms.

That said, there is a place for everyday words about mental states in the psychological and brain sciences. They are the coin of the mental realm, and are necessary when talking about our mental lives, even scientifically [51,57,59,60]. Summarizing such views about the special role of common language in psychology, I wrote this in my 2015 book *Anxious* [47, p. 40]:

Physicists, astronomers, and chemists don't need to take seriously commonsense ideas about nature because people's beliefs and attitudes about the stars, matter and energy, and chemical elements don't affect the subject under investigation. The fact that we commonly say (and some may actually believe) that ‘the sun rises in the east’ does not have any scientific bearing on the fact that sunrise is an illusion. But psychologists do have to pay attention to folk psychology because people's common beliefs about the mind influence their thoughts and actions in daily life and are thus an important part of what psychology is all about. Folk psychology is a window into the things that interest people and affect their lives.

## 7. Deep survival

Amygdala defensive survival circuits are present throughout vertebrate species [61–64]. One proposal is that this is a derived trait that emerged in fish tens of thousands of years after the first fish arrived [65,66]. Another possibility is that early vertebrates, in fact, had an amygdala homolog. That this may be the case is suggested by the work of Sten Grillner and colleagues on the jawless fish, lamprey, one of the oldest living vertebrates [67,68]. In fact, their findings show striking similarities between the entire forebrains of lamprey and all other vertebrates. Recent findings showing that the vertebrate telencephalon evolved from a brain precursor region in ancestral chordates implies homology of these structures. Given this, it is possible that the amygdala, a region of telencephalon, may also have originated from this precursor region [69–71].

Most invertebrates, though, belong to a separate line of descent. Although their nervous systems are not equipped with an amygdala, they do behave defensively, and have their own defensive survival circuits. For example, circuits have been identified that control freezing in flies [72]. This finding was unfortunately described as being functionally similar to ‘fear’ circuits in mammals and, as such, suggested that it may be able to shed light on aspects of human ‘fear’. This kind of prose invites the conclusion by journalists that flies may be emotional beings with conscious feelings of fear similar to ours [73]. This is an excellent example of how using the survival circuit terminology could have helped avoid unintended misrepresentation in the press of otherwise important research.

Consistent with the idea of conserved survival circuitry in diverse animals is the fact that, as mentioned above, some genes and molecules involved in implicit learning about and control of behaviours in the presence of threat are conserved in vertebrates and invertebrates. This does not necessarily mean that having the genes shows that the function is conserved. But the correlation between the presence of the genes and the function is at least suggestive that similarities in implicit forms of learning in vertebrates and invertebrates may be due to a common set of synaptic plasticity-related genes inherited from a common basal ancestor. Research using modern genetic tools, in fact, supports this suggestion [74–76].

But the connection runs deeper. Some of these plasticity genes and molecules are present in radial organisms with diffuse, poorly centralized, nervous systems, like jellyfish and hydra, which express simple forms of learning [77]. Some of these genes and molecules also exist in sponges, which lack nervous systems, but may, long ago, have had one [78]. However, the focus on inherited features of nervous systems may be the wrong emphasis, as the trend predates neurons and synapses.

Single-cell protozoa share a common ancestor with animals. Being unicellular, protozoa could not have ever had a nervous system [78–81]. Yet they behave, approaching useful and avoiding harmful stimuli [82]. This is at least superficially similar to animal behaviour. Also like animals, they undergo simple forms of implicit learning about biologically significant stimuli [83], and possess some of the same plasticity genes and molecules that animals have [74–76]. And even bacteria approach useful and avoid harmful stimuli [84] and may even be capable of simple implicit learning [85], but this is not well established.

The key takeaway, though, is not about learning. It is about behaviour. Darwin's protégé Romanes treated behaviour as an ambassador of the mind [86]. While this, of course, is true to some extent, behaviour is not fundamentally a psychological capacity [1,2]. It is simply one of the tools that organisms use to go about the day-to-day business of staying alive.

Bacteria and protozoa, for example, do not approach food out of hunger or ‘tumble’ away from harmful molecules out of ‘fear’. Such responses are defined by the physical fact that any movement of an object places it further from or closer to other objects in the environment, and by the physiological fact that such movements support survival in harmful and beneficial situations, respectively.

If withdrawal and approach are universal, life-sustaining capacities of all extant organisms [87], it follows that such behaviours may likely have existed as part of the homeostatic survival toolkit of early cells, which likely had to move around to manage their physiological viability in an ever-changing milieu—as soon as there was life, there was danger.

My inclination after reaching the above conclusions was to assume that animals inherited fundamental survival behaviours related to defense, feeding, fluid regulation and reproduction from unicellular microbes by virtue of genetic and molecular inheritance. But I realized there was another, less biologically daunting, way to think about this than to assume that complex defensive, feeding and reproductive behaviours of present-day animals were literally inherited from ancient microbes.

All organisms have to manage energy resources, regulate intracellular fluids, defend against harm and reproduce, but the way any one organism does these things depends on the kind of body its species evolved. For example, different mammalian species flee from danger by running, flying or swimming, depending on the kind of locomotory capacities that evolved with their bodies. In short, behaviours are bauplan-dependent (species-specific) survival implementations of universal (species-general) survival requirements. And survival circuits are the species-specific neural implementation by which the behavioural implementation of the universal requirement is achieved in each animal.

The point is, we should not be looking for how motor features of defensive or feeding behaviours were passed on from bacteria to archaea, and from them to protozoa and from them to animals. How animals manage survival motorically is essentially an artefact of their species' body plan. Therefore, what we need to understand are the physiological substrata of survival functions that have been maintained across evolutionary transitions to ensure that animals possess body plan-appropriate, life-sustaining motoric responses that sustain individual life.

## 8. Human emotional experience

If the conscious mental state of fear in humans is not a product of the amygdala defensive survival circuit, how does it come about? I propose that it comes about much like any other conscious mental state. Although there are a number of theories of consciousness [88–93], I will focus on my cognitive account of mental state consciousness [2,94–98].

**Figure 2.**
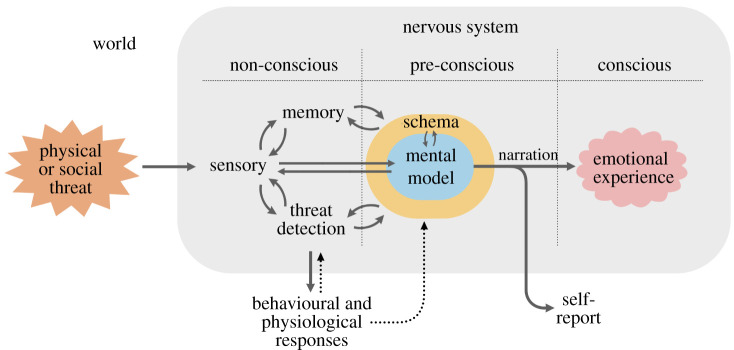
My cognitive model of the conscious experience of fear in humans. Reproduced from [96]. (Online version in colour.)

It is sometimes said that emotions like fear are universal. But I argue that it is danger, not fear, that is universal [19,94,96]. I define fear as a personal, schema-based, narrative-driven, culturally shaped, subjective experience that occurs in a biologically or psychologically significant situation (figure 2). Schema are collections of memories about specific kinds of situations in life that underlie our thoughts, feelings and actions in life.

The key idea is that conscious experience, whether emotional or not, is always preceded by non-conscious (pre-conscious) cognitive processing of sensations and memories, including schema, that results in a mental model of the situation. In an emotional situation, such as fear, brain and body consequences of threat-elicited survival circuit activation become part of the model. The output of the model is a non-conscious narration (which can be verbal and/or non-verbal). The narration then supplies the content of the conscious experience, and separately, the content of verbal report. Because conscious experience and verbal report involve different downstream circuits, verbal report does not always perfectly reflect the person's experience.

Nevertheless, verbal report is the most reliable means, the gold standard, for assessing consciousness in humans [99–102]. The importance of verbal report was recognized in the late nineteenth century by the pioneering brain scientist David Ferrier, who lamented the absence of verbal report in monkeys and the limits that this lack imposed on his ability to study conscious perception in them [103]. The use of verbal report in studies of human consciousness will be discussed further below.

This perspective on emotion, of course, relates back the conclusion that Gazzaniga and I drew from our studies of split-brain patients—that an important feature of human consciousness is the maintenance of a sense of mental unity via cognitive interpretations that attribute meaning and cause to behaviours that are controlled non-consciously by the brain. In extending our ideas to emotional consciousness, we built on Schachter and Singer's cognitive theory of emotion [104], which I continue to do in spirit, if not substance, as do many other current cognitive theories of emotion [105–111].

A popular cognitive approach theory of emotion takes a constructionist perspective [105,112,113]. My approach falls roughly into this camp to the extent that it treats conscious emotions as cognitive conceptions that are assembled in the moment, rather than being elicited as innate mental states. I was on this track long before it carried the constructionist label.

The typical constructionist perspective views the conceptions underlying emotions as the result of interactions between two generalized activities in the brain, valence and arousal, and minimizes the importance of innate, species-typical processes. This reflects constructionists' battles with the so-called basic emotions theories, which treat core emotions like fear—including the conscious experience of fear—as innate products of subcortical circuits [35]. My model falls between basic and constructionist theories.

Like basic emotions theories, I emphasize innate circuits (in my case defensive survival circuits rather than fear circuits) that control behavioural and physiological body responses elicited by threats. But unlike basic emotionalists, and like constructionists, I view the conscious experience of fear as a product of cortical, cognitive activity. Unlike traditional constructionists, though, I think of arousal and valence as activities triggered by the particular innate survival circuit that is active at the moment. For example, arousal, rather than being a purely generalized activity that occurs in any biologically significant situation, has elements that are tailored to current circumstances by the active survival circuit. Donald Pfaff has argued for something similar in relation to arousal—that it has specific and generalized components [114].

Another way my model differs from constructionist and other cognitive theories of emotion is that I have situated it within the higher-order framework of consciousness [88,90,115]. This class of theories proposes that conscious experiences result from the higher-order re-representation, hence conceptualization, of lower-order information. In the brain, the hypothetical higher-order network involves regions of lateral prefrontal cortex (PFC), including dorsal and ventral lateral PFC and the lateral frontal pole. For example, the conscious experience of an apple, in this theory, reflects the lateral PFC re-representation of lower-order visual cortex states.

**Figure 3.**
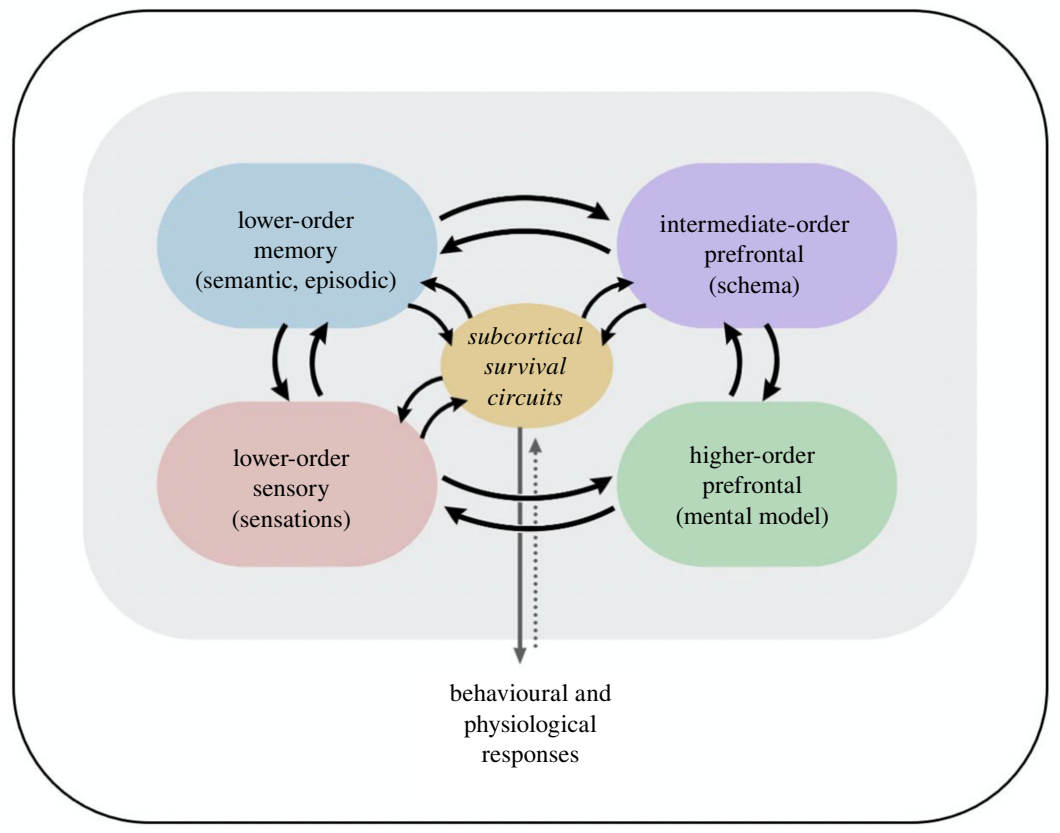
Multi-state representation in higher-order awareness. Reproduced from [96]. (Online version in colour.)

Contemporary theories of consciousness, including higher-order theories, seldom consider the role of memory in consciousness. But without semantic memory, and especially memory-based schema [116,117], the sensory representation of an apple is not meaningful as an instance of the fruit of that name [118]. My multistate representation model makes memory an essential underpinning of higher-order consciousness, including emotional consciousness [94,96–98] (figure 3).

In the brain, for memory to impact visual perceptual consciousness, the activity of temporal lobe memory systems must be integrated with the sensory signals. A key way this occurs is by way of interactions between sensory cortex and temporal lobe areas, especially medial temporal lobe regions such as the hippocampus and perirhinal cortex [119]. Schema, in fact, are believed to be assembled and used by way of hippocampal interactions with medial PFC [117]. These sensory–memory representations, including schema, are then further represented by connections with prefrontal cortex areas, including the orbital, ventral medial and anterior cingulate areas located in the medial wall of the hemispheres, and the insula cortex buried deep in the Sylvian fissure. Each of these, in turn, interact with lateral PFC areas (dorsal and ventral latera and frontal pole), by way of various intra-PFC connections [120], as discussed in detail elsewhere [2,90,94,96]. In the context of my higher-order theory, the medial wall and insula areas are considered ‘intermediate’ between sensory/memory circuits and lateral PFC areas. These intermediate areas have been implicated not just in memory re-representation, but also in the encoding of body state signals, stimulus and response values, and self [94]. In the view presented here, such representations are not sufficient, on their own, and must be further represented by lateral PFC for the kind of higher-order awareness we humans experience. But this is hypothetical, and a higher-order account based on the intermediate areas is also imaginable.

If human emotions are cognitive processes, what makes them different from non-emotional cognitive states? Quite simply, the higher-order network re-represents additional lower-order information when the stimulus is an emotional one. For example, in fear, the consequences of amygdala defensive survival circuit activation in the brain (i.e. arousal) and body (visceral and somatic signals that feed back to the brain) are often also re-represented in the medial/insula PFC areas mentioned above. Aspects of the various prefrontal representations are then further integrated in the lateral PFC as a higher-order re-representation of diverse lower-order states. The emotional experience, therefore, depends not just on PFC but also on the entire cascade of non-conscious sensory, mnemonic/conceptual and survival circuit processing that is antecedent to PFC areas. It bears repeating that every conscious state is, until its last moment of fruition, non-conscious.

Other primates have similar, though less developed, lateral and medial/insula areas, while non-primate mammals mainly possess the medial/insula areas [2,96,121]. This anatomical situation may limit the kinds of conscious emotional states each can assemble relative to humans [96], as elaborated later.

The reason that amygdala survival circuits are so often associated with fear is because of their prominent role in controlling defensive responses when humans or other animals are in danger of bodily harm from predators. As we have seen, this brain function runs deep in the vertebrate lineage. However, predator danger is only one of the many kinds of situations that can cause fear. If you are trapped on a mountain top without food, water, or shelter, body signals indicating low reservoirs of nutritional/energy supplies or fluids, or low body temperature, can lead to fear of death by starvation, dehydration or hypothermia. In humans, fear can also arise from being near someone with an infectious disease, or the news that you may have a life-threatening condition, or from family stress, social abuse, job insecurity, political instability or just about anything imaginable—threats do not have to be real to make one feel afraid or anxious. The only way to account for all of these under a single concept of fear is by way of cognitive interpretation of the situation in which you find yourself, or that you imagine you could be in. Innate states just would not do the trick.

## 9. The problem of animal consciousness

The question of how emotions come about in the brain of animals is subset of the thorny question of animal consciousness. I have written extensively about this [2,42,44,46,47,96] and can only touch on some of the key points here.

The question of whether non-human organisms are conscious, including mammals, lower vertebrates and invertebrates, and if so how, is quite contentious. The issues are far more complex than trying to find evolutionary roots of behaviours. We can directly measure behaviour in present-day organisms to obtain clues about what kinds of behaviours may have been present in their early representatives, and in the common ancestors they share with other organisms, in much the same as we do with brain structures. Consciousness, though, is more challenging. Whether even our closest primate relatives are conscious is debated [122,123]. The problem is that behaviour and physiology have limits as measures of consciousness [124], especially when only non-verbal behaviour is available, as discussed further below.

In the late nineteenth century, animal psychology tried to use intelligent and emotional behaviour as marks of consciousness [86]. This, in part, is what led to behaviourism. With the behaviourists gone, some animal psychologists have revived such efforts. While the behaviourists certainly went too far, they were quite effective, if too dogmatic, in managing the language of psychology. And with them gone, we are back to the semantic ‘wild west’ of the late nineteenth century.

Part of the problem is that the evaluation of consciousness in animals, whether in mammals [35] or molluscs [125], is often based on intuitions and beliefs derived from one's understanding of their own behaviour, mind and/or brain. When the claims match common sense and lore, they feel correct, and when they are repeated authoritatively in scientific or lay communities, they come to be assumed as indisputable facts. This does not mean that other animals are unconscious robots. It simply means the behavioural data held up as proof of mental states are often not. The widespread assumption that innate defensive behaviours are a fool-proof reflection of conscious feelings of fear is a case in point.

According to J. S. Kennedy, author of *The new anthropomorphism*, ‘Anthropomorphic thinking … is built into us … It is dinned into us culturally from earliest childhood. It has presumably also been ‘pre-programmed’ into our hereditary make-up by natural selection, perhaps because it proved to be useful for predicting and controlling the behavior of animals' [126, p. 5]. Our language, he says, is inherently anthropomorphic, and, as a result, our concepts and thoughts tend to lean in this direction as well. Kennedy concludes that anthropomorphism is part of human nature. This is perhaps why we all see human-like emotions in our pets. Me included. We do not have to be rigorous scientists every moment of waking life—but we must when we are being scientists.

At the turn of the twentieth century, when anthropomorphic approaches were commonplace in comparative psychology, Herbert Spencer Jennings, who studied the behavioural repertoire of single-cell protozoa, put it this way: ‘If *Ameoba* were a large animal, so as to come within the everyday experience of human beings, its behavior would at once call forth the attribution to it of states of pleasure and pain, of hunger, desire and the like’ [82, p. 336]. But beyond such general tendencies toward anthropomorphism, each scientist also comes to his or her career with personal biases and dispositions. As the philosopher Bertrand Russell once noted, ‘All the animals that have been carefully observed have behaved so as to confirm the philosophy in which the observer believed before his observations began’ [127, pp. 29–30].

Often, proponents of animal consciousness argue by analogy with human behaviour. This has been criticized by many on the grounds that it tends to ignore ‘leaner’ alternatives that explain behaviour without recourse to consciousness [122,123]. The critics do so, not to deny consciousness in non-human animals, but in the spirit of fostering a more rigorous scientific evaluation of what the data allow us to conclude about consciousness on the basis of behaviour alone.

Problems especially arise when the claims, rather than being treated as opinions or hypotheses, are presented as indisputable facts that are presumed to be so obvious that no reasonable person could question them. And those who show interpretative restraint are denigrated as ‘deniers' [128], a term that has a variety of negative connotations in contemporary culture, and that is not suitable in a scientific debate.

Similar issues exist when evaluating consciousness in preverbal children. Just because an infant human, or a rat, bird, lamprey, amphioxus or octopus, behaves to an environmental stimulus in a way that an adult human might when they are conscious of a similar kind of stimulus does not *necessarily* mean that the child or animal is having a conscious experience, a mental state, similar to what the adult human has. They *may,* and in some cases, likely, have some kind of conscious experience. But how can we truly know if they do, and if they do, what it is like for them?

To get around this conundrum one often has to make compromises about what is acceptable as scientific support for the hypothesis. At the end of the day, claims of consciousness in animals and human infants often rest more on intuitions and beliefs than on data, since there are often equally compelling non-conscious explanations of the behaviour [122,123].

Why are we on any firmer ground studying consciousness in human adults than in adult animals? Two points are crucial. One is that barring a congenital brain disorder, all humans have brains with same basic structural components and functional capacities. Given that, if I am conscious of my mental states and actions, I can with some confidence assume that you have this capacity as well. Like any species trait, consciousness will vary across individuals, but will be present, or at least is potentially present, in all members of our species.

The second point is that we humans can report on our conscious sates verbally or non-verbally, but can only report on non-conscious states non-verbally. Other animals, and human infants, lacking the ability to verbally report, only have non-verbal means of responding to both conscious and non-conscious states. There is, therefore, no easy way to distinguish conscious and non-conscious states using non-verbal behaviour alone. Given the points I made above about the shortcomings of behaviour as an index of consciousness, even in humans, we have a significant methodological barrier. Obviously, the importance of verbal report as a methodological tool should not be taken to mean that human infants or language-impaired adults or non-human animals lack consciousness.

## 10. An empirical approach to animal consciousness

To be clear, even if repetitive, I do not deny consciousness in other animals. I am perfectly comfortable saying that some animals likely have conscious experiences, despite the methodological barriers that prevent clear proof. Yet, even if they are conscious, they would not be conscious in the ways we are. After all, human bodies differ in unmistakable ways from the bodies of other animals, including the bodies of our closest primate relatives. Similarly, our brains differ in important ways from the brains of other primates cousins [129–132], and theirs from other mammals [121,133]. It should, therefore, not be controversial to suggest that their mental states, including their emotional states, may also differ from ours.

Yet, in the light of the methodological issues, how would we ever know what the experiences of other animals are like? One way might be to use understanding of conscious experiences and their representation in the human brain as a basis of understanding what kind of consciousness other animals *might,* and *might not*, possess, given the ways that their brains are like, and different from, ours. I explored this idea in relation to other primates and other mammals in a recent publication [96] and will summarize and extend these suggestions below.

It is important to start by pointing out that the word ‘consciousness' is often used as if it has a single referent. But it does not, and this has caused much confusion. At a minimum, it is important to distinguish ‘creature consciousness’ from ‘mental state consciousness' [88] (table 1). The former refers to the condition of being awake and behaviourally responsive to environmental stimuli. All bilateral animals have creature consciousness. Mental state consciousness, on the other hand, is the condition of actually experiencing the world and one's relation to it using mental models. It is crucial to keep this distinction in mind when evaluating claims of consciousness in animals, as some claims are primarily about creature consciousness.

**Table 1.** Partitioning consciousness.

Creature Consciousness (the state of being alive, awake, and behaviourally responsive to sensory stimuli)
Mental States Consciousness (inner awareness of sensations, thoughts, feelings, and/or actions)
— Autonoetic Consciousness (cognitive awareness of oneself as an entity with a past, present and possible future)
— Noetic Consciousness (cognitive awareness of facts and/or ideas about the world and oneself
— Anoetic Consciousness (non-cognitive, fringe awareness of external stimuli or internal states)

For example, a much cited paper by Merker [134] defined consciousness as the state of being awake and responsive to sensory stimuli (i.e. creature consciousness). He used findings from patients with limited cortex due to hydrocephaly, and decorticated rats, as core components of his evidence. In these instances, and others he cites, consciousness is judged by sensory–motor responsiveness, not by evidence of mental states. In response to commentaries on his views, he says he is not interested in adult human consciousness (presumably cognitive-based mental state consciousness) but something more primitive. This implies that here he is referring to some kind of primitive mental state rather than creature conscious. But what would this be? Perhaps he meant a form of sensory sentience, which Lacalli proposed may have even existed in the invertebrate chordate ancestors of vertebrates [70,71]. I will build on a partition offered by Endel Tulving [135] that I have used in recent publications [2,94,96–98]. It may be able to account for what Merker might have had in mind, but in relation to mental state consciousness.

Tulving distinguished between three forms of mental state consciousness in humans (table 1). These are autonoetic (explicit self-awareness of one's existence over time), noetic (explicit awareness of facts and concepts about the world or one's self) and anoetic (implicit awareness of the world) [2,94,96–98,135,136]. The first two are cognitive (i.e. based on episodic and semantic explicit memory) and the third is procedural (i.e. based on implicit learning). Another way of saying this is that autonoetic and noetic consciousness depend on the use of internal representations and mental models, but anoetic consciousness does not [96].

Because anoetic consciousness is not an intuitive idea, two clarifications are in order.

First, although ‘procedural’ and ‘implicit’ traditionally imply 'non-conscious’ states, in the case of anoesis, non-conscious does not mean completely unconscious. Anoetic states reside on the ‘fringe’ or ‘penumbra’ of the ‘stream of consciousness', to use William James’ terms [137], where the line between conscious and unconscious is fuzzy. In humans, these states are typically overshadowed by cognitive consciousness and go unnoticed [136]. But they are nevertheless present, and noticeable when cognitively attended to. They co-occur with cognitive states of consciousness and give these a feeling of ‘warmth’ and ‘intimacy’, again borrowing from James. They underlie your ability to know that your mental and body states are yours, without you ever having to explicitly affirm this [96,98,138]. The second clarification is that Tulving treated anoetic consciousness as a primitive awareness about the external world. However, I [96] and others [136] have proposed that penumbral/fringe anoetic states also represent the internal milieu (i.e. physiological conditions of the body and brain). Such anoetic states may be what Merker had in mind when he referred to a primitive kind of consciousness that is different from adult human consciousness.

The reason I am devoting so much discussion to Tulving's partitions of conscious mental states is because I believe that understanding their representation in the brains of humans might give us insights into the processes the underlie mental state consciousness in other animals, especially those in our evolutionary past [2,96]. In particular, although we cannot directly measure consciousness in other animals, Tulving's three states offer an indirect way in—because the three states depend on behaviourally measurable episodic, semantic and procedural memory processes, these provide pre-conscious proxies that can be studied in non-human species.

Researchers have used behavioural measures of episodic memory to ask whether primates, rodents and birds might have this capacity [119,139–142], which is so prominent in humans. Such behavioural studies can thus test the cognitive underpinnings of autonoetic consciousness in animals, but cannot measure autonoetic consciousness itself. To acknowledge this, the term ‘episodic-like’ memory is often used [140]. The key difference is that episodic/episodic-like memory is about what happened and when and where it happened, while autonoetic consciousness is about reflective awareness of one's self over time in such episodes. Episodic-like memory, in short, takes researchers all the way to the finish line of consciousness. This is a significant achievement, despite not being able to cross the line.

Similarly, semantic memory has been well researched in primates and other mammals. For example, studies of object recognition in relation to instrumental-goal directed behaviours test what animals cognitively know factually and conceptually [119,143–146]. Procedural memory has also been extensively studied in primates and mammals [26,119,147]. As with episodic memory, studies of semantic and procedural memory tell us about the foundation of noetic and anoetic awareness, but do not demonstrate that the awareness itself exists.

Tulving's partitions thus offer a top-down (reverse engineering) approach that starts with human consciousness, as opposed to bottom-up (forward engineering) evolutionary approaches to animal consciousness that start in the distant past [35,70,71,125,136]. An advantage of the top-down approach is that it explicitly identifies how consciousness might differ in different animals in our evolutionary past relative to our kinds of consciousness, based on similarities and differences in their brains and ours. In other words, our kind of consciousness tells us what to look for. Clearly, the further back we try to look, the harder it gets to use human consciousness to understand possible states of consciousness in other animals. But given the striking similarities in the detailed organization of the forebrain in all vertebrates [67,68,148], and the known involvement of homologous circuits in the relevant learning processes, we have some basis for speculating about the underpinnings of consciousness.

In humans, much is known about the neural basis of explicit (episodic and semantic) and implicit (procedural) memory [26,119,149–152]. The brain areas involved in the encoding of episodic memories include regions of the hippocampal formation, regions of medial PFC (anterior cingulate and ventromedial PFC) and regions of lateral PFC (dorsolateral and polar PFC). For semantic memories, including schema, the lateral prefrontal areas involved are the same as in episodic memory, but some areas of the hippocampal formation differ (parahippocampal in episodic; perihinal in semantic), and some areas of medial PFC also differ (anterior cingulate in episodic; media orbital in semantic). The ventromedial PFC then connects both anterior cingulate and media orbital to lateral PFC areas. Note that these are not simply one-way cascades, as there are reciprocal connections at each step.

Implicit procedural memory does not involve a single system, but instead is stored in circuits that process stimuli and/or control responses. For example, plasticity in sensory and motor cortex in sensory–motor learning; the amygdala in Pavlovian threat conditioning; and the basal ganglia in instrumental habit learning [19,26,153–157].

Non-human primates have homologs of many the areas implicated in episodic and semantic memory, with the possible exception of the lateral polar PFC [130,158,159]. At a minimum, this allows primates to use non-verbal episodic and semantic memory (including schema) to create mental models and use these to guide responses to recognized objects. Similarly, procedural circuits are quite similar in in non-human primates and humans.

Other mammals possess homologues of the medial areas involved in non-verbal episodic and semantic memory present in primates and humans, but lack homologs of lateral PFC areas [121,133]. They thus must depend on hippocampal formation interactions with medial PFC for memory re-representations. Procedural circuits in mammals are largely homologous with those in non-human primates and humans.

The interim conclusion from this discussion is that the three kinds of memory/learning processes discussed reflect phylogenetic elaborations of the PFC over the evolutionary history of mammals. The implication is that the three kinds of conscious states associated with these memory processes may have followed the same evolutionary course, either arising in tandem with the memory/learning circuits, or because of their existence. However, extension to consciousness remains hypothetical.

Can we take these ideas to lower vertebrates? An entry point for this may be the suggestion by Shepherd and others that, in mammals, olfactory paleocortex, via connections with medial wall cortical areas, especially orbitofrontal cortex, plays a role in olfactory consciousness [160–162]. But what kind of consciousness might that be? Following Tulving's scheme, it can be called anoetic olfactory consciousness. Also of note is that olfactory paleocortex and orbitofrontal cortex are both connected with the amygdala in mammals. This suggests the possibility that orbitofrontal re-representation of olfactory-triggered amygdala states might constitute anoetic experiences about the biological significance of olfactory stimuli, and of the bodily consequences that co-occur with these significant stimuli. But diurnal mammals also rely on other senses, and these also activate the amygdala, allowing a wider range of anoetic states via cortical re-representation.

Early vertebrates possessed a medial pallium, a primitive homologue of the mammalian hippocampus, that received olfactory and visual inputs and created and stored spatial representations that guided navigation and foraging [148,163–165]. If the amygdala homologue in early vertebrates was also connected with the medial pallium, re-representation of sensory and amygdala activity by the medial pallium may have supported both olfactory-based anoetic sensory experience, and amygdala-based anoetic experience of biologically significant stimuli. Given that they lack medial PFC homologues, medial pallium re-representation of sensory stimuli would have been the sole basis for sensory and amygdala informed experiences. As a result, such a state would likely have been even more primitive than anoetic experiences in mammals.

If the above is roughly correct, it would have an important implication. Namely, the medial pallium's capacity to acquire and retain spatial maps may have paved the way in mammals for additional functions of the hippocampus. In particular, I have in mind its capacity to form and store complex semantic memories and schema, and, via re-representation in medial PFC, to create mental models that underlie non-verbal noetic conscious awareness of the meaning of stimuli and responses in light of their value in survival situations. In non-human primates, and the evolution of lateral PFC, more elaborate executive functions may have enabled more complex forms of non-verbal behavioural control and noetic awareness. Further expansion of lateral PFC in humans, including enhanced executive functions and top-down control, and the invention of language, may have ushered in verbal noesis and autonoesis.

So far, I have described how different kinds of consciousness may have evolved in vertebrates. But this discussion begs the question of what might be the functional advantage of consciousness? The most general answer is that consciousness opens up novel forms of behavioural control [166]. But let me be more specific. Following the evolutionary thread just discussed, in early vertebrates, non-cognitive anoetic consciousness may have provided important advantages in navigation and foraging, including novel processing capacities for sensing the world, learning relations between sensory processing and biologically significant stimuli, and selection of actions based on past consequences [167]. With cortical expansion in mammals, and then primates, additional survival advantages related to behavioural control may have come with more complex anoetic and novel cognitive (noetic) consciousness, with the latter involving the use of mental models. And in humans, with still further cortical expansions and the addition of language, verbal-based cognitive noesis and autonoesis have allowed us to know ourselves as entities with a past and present, and also to anticipate possible futures. Regardless of whether actual states of consciousness can be experienced in non-human animals, the evidence described shows the kinds of neural underpinnings that may underlie the evolutionary past of anoetic, noetic and autonoetic consciousness in humans, and perhaps in some non-human animals as well.

These ideas are, of course, highly speculative. But by linking the speculations to facts about the structure and functions of the human brain, and following the evolutionary history of the relevant structures underlying different kinds of conscious experiences in humans back in history, we may have at least some empirical grounding for such speculations.

## 11. Mental disorders put things in perspective

One of the factors that has motivated my efforts to reconceptualize fear is the much-discussed failure of the pharmaceutical industry to find better treatments for problems related to fear and anxiety [168,169]. The typical approach to drug discovery is to test animals, often rodents, using ‘fear-like’ or ‘anxiety-like’ behavioural tasks and administer compounds to them. The assumption is that because we inherited our ‘fear’ circuit from our mammalian ancestors, and because this circuit both assembles feelings of fear and controls behavioural and physiological responses in danger, it should be the case that drugs that make rats less timid behaviourally and less physiologically aroused should make people less fearful or anxious.

Yet, despite decades of research, new, better treatments were seldom found [170]; drugs that showed promise in animals often flopped in human clinical trials [171], and while those that made it into circulation may have had fewer side effects, they were at best only as effective as the older ones. As a result, Big-Pharma has been withdrawing funding for research on fear and anxiety, and also depression [168,169].

Given the various points made earlier, it seems unrealistic to have expected that drugs that change the behavioural or physiological responses in rodents would be the solution to the problem of how to make people feel subjectively less fearful or anxious. The more realistic expectation is that behavioural (avoidance) and physiological (hyperarousal) symptoms might be dampened, since that's what we know with confidence the drugs did in the laboratory studies. But if an anti-anxiety medication makes you less jittery and avoidant, but you still feel anxious, the treatment is not living up to the promise of its name.

To be fair, some drugs give some people some psychological relief. But the question is, does this result from the medication turning off a fear or anxiety circuit in the brain, or is it because it has indirect effects on feelings of fear or anxiety? Specifically, the psychological relief, when it occurs, could be secondary to other changes that affect fearful and anxious feelings. For example, some relief could come from alteration of defensive survival circuits that control behavioural and physiological symptoms, and/or alteration of somatic and visceral motoric control circuits in the brainstem, the spinal cord or the periphery that are the final paths to the symptoms.

Clearly, such indirect effects are useful to the patient. And if changing such symptoms was the expectation, the industry, treatment community and patients might view the medications as a rousing success, since they would be understood as doing what they were designed to do in the animal studies, rather than not doing what they were incorrectly advertised to do. Another possible account of positive effects, at least for medications like benzodiazepines, on anxious feelings is general emotional blunting, rather than a specific reduction in anxiety, resulting from sedative effects consequent to enhancing GABAergic inhibitory neural activity in widespread areas of the brain [172].

It matters that we figure out how treatments work. Otherwise, we will never advance the cause of finding the best treatment, given the patient's particular problems. The fact is, what people really want from any kind of therapy is to feel better subjectively. The best way to improve this likelihood would be to put the mental back into mental disorders [48,173,174].

## 12. Conclusion

The survival circuits of mammals are neural implementations of ancient and necessarily persistent physiological survival requirements that have allowed organisms throughout the history of life to move in ways that extended their life beyond the present moment in biologically significant situations. The consequences of survival circuit activation can indirectly modulate conscious feelings. But they do not, on their own, define the conscious content of feelings. Feelings, instead, are cognitive interpretations of significant situations that we encounter in life. Although I had some inkling of these conclusions decades ago from studying split-brain patients, it was my decades of work on rodents that allowed me to see the field the way I do today. The approach I offer here, based on a distinction between ancient defensive survival circuits that control behavioural and supporting physiological responses, and more recent cognitive circuits that assemble conscious feelings of fear, seems at least worth considering as a possible way forward.

The bottom-line general conclusion I would like to end with is that our understanding of a psychological process in the brain is only as good as our conceptualization of what that process is. If we do not know what we are looking for, we will never find it. And as we search for knowledge about psychological processes in the brain, we should only use mental state words to refer to mental states. Additionally, we should refrain from attributing behavioural control to mental states, whether in humans or other animals, without compelling evidence. It is of course acceptable to speculate about the mental lives of other animals, which I have done my share of in this article. But when we speculate, we should be clear that we are doing so. I hope that I have at least achieved this.

## Data accessibility

This article does not contain any additional data.
